# Recent advances in mechanisms of food allergy and anaphylaxis

**DOI:** 10.12688/f1000research.25638.1

**Published:** 2020-07-31

**Authors:** Sunil Tomar, Simon P Hogan

**Affiliations:** 1 1. Mary H. Weiser Food Allergy Center, Department of Pathology, University of Michigan 4051-BSRB, 109 Zina Pitcher Place, Ann Arbor, MI, 48109-2200, USA

**Keywords:** Anaphylaxis, Food allergy, Oral tolerance, Allergic sensitization, Type-2 immune responses, Microbiota, Antigen sampling, secretory epithelial cell antigen passages, IL-9 producing mucosal mast cells, Oral immunotherapy, Clinical trials

## Abstract

Food allergens are innocuous proteins that promote tolerogenic adaptive immune responses in healthy individuals yet in other individuals induce an allergic adaptive immune response characterized by the presence of antigen-specific immunoglobulin E and type-2 immune cells. The cellular and molecular processes that determine a tolerogenic versus non-tolerogenic immune response to dietary antigens are not fully elucidated. Recently, there have been advances in the identification of roles for microbial communities and anatomical sites of dietary antigen exposure and presentation that have provided new insights into the key regulatory steps in the tolerogenic versus non-tolerogenic decision-making processes. Herein, we will review and discuss recent findings in cellular and molecular processes underlying food sensitization and tolerance, immunological processes underlying severity of food-induced anaphylaxis, and insights obtained from immunotherapy trials.

## Anaphylaxis

Anaphylaxis is a serious and life-threatening generalized or systemic allergic or hypersensitivity reaction which is rapid in onset (minutes to a few hours) ^1, 2^. Anaphylaxis diagnosis is based upon the involvement of at least two organs system including skin/cutaneous, gastrointestinal (GI), respiratory, cardiovascular, and neurologic systems ^2– 5^. While foods remain the most common cause of anaphylaxis, other causative agents include medicines and insect stings ^2^.

Recently, the European Academy of Allergy and Clinical Immunology (EAACI) and the American Academy of Allergy, Asthma, and Immunology (AAAAI) published a consensus document to propose a new approach to personalized treatment for patients of food allergy, drug allergy, and anaphylaxis ^2, 6^. The personalized medicine approach, termed precision medicine, would be guided by underlying cellular or molecular mechanisms, termed endotypes, and associated diagnostic biomarkers rather than the clinical presentation of clinical symptoms of anaphylaxis, termed phenotype ^2, 6^. It is anticipated that better clinical outcomes would be achievable if treatments were tailored according to the specific cellular/molecular characteristics (endotype) rather than clinical characteristics of a patient (phenotype) ^6^. Two recent reviews have described the phenotypes, endotypes, and biomarkers to aid the diagnosis of anaphylaxis in great detail ^2, 6^. Proposed anaphylaxis endotypes include immunoglobulin (Ig) E-mediated and non-IgE-mediated type I reactions, cytokine release reactions, mixed reactions, and complement or bradykinin-mediated direct activation of mast cells (MCs) and basophils ^2, 6, 7^. The development of precision medicine for anaphylaxis is going to be reliant on future research in the underlying cellular and molecular processes that drive food sensitization and anaphylactic reactions and the identification of specific biomarkers to predict anaphylaxis endotypes, severity of reaction, and clinical outcome with treatments ^6^. Herein, we will describe recent advancements in our understanding of the underlying immunological processes that regulate oral tolerance versus food sensitization, mechanisms of dietary antigen sampling, severity of anaphylactic reactions, and oral immunotherapy outcomes.

## Oral tolerance versus sensitization

To prevent the development of systemic immune responsiveness to innocuous dietary protein antigens, the immune system has developed mechanisms of local and systemic immune unresponsiveness termed “oral tolerance” ^8– 10^. Luminal soluble dietary protein antigens are acquired by small intestine (SI) lamina propria (LP) CX _3_CR1 ^+^ macrophages and CD103 ^+^ migratory dendritic cells (DCs) that migrate via the afferent lymphatic vessels to selective duodenal gut-draining lymph nodes, where they present dietary antigens via cognate interaction to naive CD4 ^+^ T cells ^11– 13^. CD103 ^+^ DC-derived retinoic acid (RA), indoleamine 2,3-dioxygenase (IDO), and transforming growth factor (TGF)-β promote *de novo* Foxp3 expression and generation of peripheral regulatory T cells (Tregs) and expression of gut-specific homing receptors including CCR9 and α4β7-integrin ^12– 15^. The newly derived CD4 ^+^ Treg cells traffic back to the SI LP, where they undergo proliferation and maintain a tolerant homeostatic environment through secretion of the cytokines TGF-β and interleukin (IL)-10 ^16^. Tregs, specifically peripheral Tregs, and not thymus-derived Tregs, are critical for establishing oral tolerance ^16– 19^. Increased frequencies of Tregs are associated with outgrowing early on cow’s milk allergy, while lower Treg frequencies have been noted in atopic young children with food allergy ^20, 21^. The Foxp3 ^+^ CD4 ^+^ Treg cells are maintained and supported by additional regulatory cells positioned within the SI mucosa, including MHCII ^+^ CX _3_CR1 ^Hi^ IL-10-producing macrophages, gut-resident type 3 innate lymphoid cells (ILC3s), and regulatory B cells (Bregs) ^16^. Microbial-sensing intestinal macrophages secrete IL-1β to stimulate ILC3-derived GM-CSF, which supports DC secretions of RA and IL-10 ^22^. Furthermore, gut-resident ILC3s through IL-22 secretion promote enhanced barrier function and reduce permeability to dietary antigens ^23^. Bregs contribute to tolerance through the production of IL-10, TGF-β, and IL-35 ^24^. Extrathymically derived peripheral RA receptor (RAR)-related orphan receptor gamma t (RORγt) ^+^ Tregs support a protective mucosal T regulatory response and enhancement of intestinal epithelial barrier integrity ^25– 28^. Furthermore, this tolerogenic state is reinforced by protective commensal microbes and their metabolites such as short-chain fatty acids (SCFAs) (e.g. acetate, propionate, and butyrate) that bind to G-protein-coupled receptors (GPR43 [free fatty acid receptor (FFAR)-2], GPR41 [FFAR3], and GPR109A) ^29^.

In disease states such as food allergy, these tolerogenic mechanisms are thought to be dysregulated, triggering the development of a food-specific “sensitizing” IgE response that can predispose to food allergy and anaphylaxis upon subsequent food exposures. In these individuals, food allergen exposure leads to the production of the pro-type-2 epithelial-derived cytokines IL-25, IL-33, and thymic stromal lymphopoietin (TSLP) ^30^. The array of stimuli that can elicit an initial prototypic type-2 cytokine response is not yet fully elucidated. Experimental evidence suggests that dietary saturated fats such as medium-chain triglycerides (MCTs) are sufficient to promote SI epithelial-derived IL-25, IL-33, and TSLP production, driving a CD4 ^+^ T helper type 2 (Th2) response and food sensitization ^31^. MCTs are thought to induce an endoplasmic reticulum stress and unfolded protein response within the GI epithelia, leading to induction of IL-25, IL-33, and TSLP ^32, 33^. The pro-type-2 cytokines are thought to act on CD103 ^+^ DC cells to promote OX40L expression, which drives the IL-4-dependent CD4 ^+^ Th2 cells and CD4 ^+^ Th9 cells ^34– 36^, and stimulate ILC2-derived cytokines (IL-5 and IL-13), which supports the expansion of basophils and MCs and suppresses Treg function ^37^. Induction of the CD4 ^+^ IL-4 ^+^ Th2 response leads to class switching of B cells and production of allergen-specific IgE. Bone marrow and lymph node germinal centers are thought to be the dominant sites of induction of IgE ^+^ plasma cells, with the bone marrow providing the major long-term source of IgE ^+^ plasma cells in both mice and humans ^38, 39^. However, a recent study revealed that a number of gastrointestinal compartments including the stomach and duodenum are enriched for food allergen-specific IgE ^+^ plasma cells in allergic patients ^40^. Furthermore, the investigators observed clonally related IgE ^+^ and non-IgE-expressing cells in these GI tissues, suggesting isotype switching and induction of IgE ^+^ plasma cells in the GI compartment. CD4 ^+^ IL-4 ^+^ Th2 response has also been shown to promote the development of IL-9-producing mucosal MCs (MMC9s) and mature MCs ^41^. MMC9 cells are hypo-granular immature MC-like cells that perpetuate MC progenitor (MCp) maturation to mature MCs in SI via an IL-9/IL9Rα pathway ^41– 43^. Post-sensitization, upon subsequent food allergen exposure, dietary antigens cross-link the IgE bound to FcεRI on MCs and basophils, leading to the release of mediators, including histamine, platelet‐activating factor, serotonin, proteases (tryptase and chymase), and lipid‐derived mediators (prostaglandins [PGD2] and leukotrienes [LTC4, LTD4, and LTE4]), which promote the clinical manifestations associated with food‐triggered anaphylaxis ^44, 45^.

## Role of microbiota in regulating sensitization versus tolerance

Recently, a number of studies have established a strong association between changes in microbial populations within the gut microbiota and allergic/tolerogenic states and identify a role for the microbiota in barrier function and permissiveness to the development of CD4 ^+^ Th2 cells and food sensitization ^46– 50^. Clinical studies have identified that microbial enrichment for *Firmicutes*, including *Clostridium* species in children, is associated with outgrowing cow’s milk allergy, while decreased *Clostridiales* and increased *Bacteroidales* and lower gut overall microbial diversity have been associated with nut and pollen allergy in adults ^51, 52^. Increased *Enterobacteriaceae* to *Bacteroidaceae* ratio and decreased *Ruminococcaceae* abundance together with low microbial diversity were associated with decreased food sensitization in infants ^53^. Consistent with the concept that microbial communities can regulate food sensitization, transfer of microbiota from sensitized mice with increased susceptibility to food allergy (IL-4Rα gain-of-function mutation Il4Ra ^Y709F^) confers food allergic susceptibility when transferred into WT mice ^47^. Similarly, colonization of mice with intestinal microbiota from cow’s milk-allergic infants, but not healthy infants, transferred food allergic susceptibility ^54^. Conversely, the presence of the *Clostridia* species *Anaerostipes caccae* has been shown to confer protection to mice from food sensitization and anaphylaxis ^23, 54^. While the molecular basis of microbiota-mediated tolerance and sensitization is not fully understood, *Clostridia* species are thought to promote oral tolerance mechanisms via increased IgA production, expansion of Foxp3 ^+^ Tregs, RORγt ^+^ ILC3-mediated reinforcement of epithelial barrier through production of IL-22, and increased expression of antimicrobial peptides such as REG3β by intestinal epithelia cells ^23, 55– 58^. Clostridial families including *Lachnospiraceae* and *Ruminococcaceae* produce SCFAs, including acetate, propionate, and butyrate, which can mediate tolerogenic/homeostatic effects through different mechanisms ^14, 55, 56^. SCFAs can bind GPR43 and GPR109A to activate the inflammasome, increasing IL-18 production, which in turn promotes barrier function ^59, 60^. Butyrate promotes the expansion of colonic Foxp3 ^+^ Treg cells by supporting peripheral Treg generation through inhibition of histone deacetylase activity at the Foxp3 promoter ^61, 62^. Propionate signaling through GPR43 also promotes intestinal Treg expansion ^63^. The loss of microbial populations and regulatory metabolites is thought to diminish the tolerogenic immune environment, leading to impaired barrier function and increased systemic food allergens and sensitization ^50^.

Several questions remain unanswered regarding the role of microbial communities in mediating tolerance/food sensitization. How do microbial populations that predominantly reside in the large intestine influence food allergic sensitization/tolerance mechanisms which are thought to occur primarily in the SI? Is the development and sustainment of microbiota-driven tolerance dependent on individual species or larger microbial communities? Given the dynamic and complex nature of cross talk among host, microbiota, and environmental cues, long-term longitudinal studies are likely required to obtain better insight into the role of the microbiota in tolerance/allergenic sensitization.

## Food antigen sampling

The anatomical sites of dietary protein antigen uptake and mechanisms that underlie the development of the tolerogenic response and establishment of oral tolerance are not fully illuminated ^8, 9^. Given the obvious link between orally consumed dietary antigens and induction of food allergy, there has been significant focus on how dietary antigen exposure of the GI tract promotes a tolerogenic or sensitization immune response ^64, 65^. However, the demonstration of a positive correlation between environmental non-oral peanut protein exposure levels and development of peanut allergy suggests non-oral environmental exposure as a potential route of sensitization ^66^. The observed natural history of the “allergic march”, whereby individuals presenting with atopic dermatitis (AD) in infancy or early childhood proceed to develop concomitant sensitization to food and aeroallergens in later childhood and adult life, has led to the evolving concept of contribution from the skin as a route of food sensitization ^67– 71^. An archetypal example is AD, a common inflammatory skin disease in childhood affecting nearly 20–30% of the population and is associated with skin barrier disruption linked with mutations in human skin barrier genes filaggrin ( *FLG*), serine peptidase inhibitor Kazal type 5 ( *SPINK5*), corneodesmosin ( *CDSN*), and mattrin ( *TMEM79*) ^72– 74^. Early onset of AD is associated with increased risk of allergic sensitization to food allergens by 2 years of age ^75, 76^, and AD-associated risk mutations are risk factors for allergy development including peanut allergy ^77, 78^. Indeed, IgE-mediated food allergy is observed in up to 35% of children affected with AD ^79^. Importantly, neonatal skin barrier dysfunction in individuals without AD also predicts food allergy at 2 years of age ^75^. Consistent with this concept, genetic ( *Flg ^ft^, Tmem79 ^ma^*), pharmacological, or mechanical disruption of the skin barrier and allergen exposure in mice is associated with food sensitization and food allergy ^80, 81^.

An elegant study by Levya-Castillo *et al*. recently identified a link between mechanical skin injury and intestinal MC expansion and induction of food-induced anaphylaxis in mice. The authors showed that skin injury triggered systemic production of keratinocyte-derived IL-33, driving intestinal tuft-cell-derived IL-25 production and ILC2 expansion ^81^. ILC2s, through their capacity to produce IL-4, drove the expansion of SI MCs, potentiating the effector phase response ^81^. Consistent with the concept of disruption to the cutaneous layer and expansion of intestinal MC populations, the authors demonstrated that duodenal MCs were expanded in AD patients ^81^. Collectively, these studies identify a direct mechanism whereby epicutaneous allergen exposure and skin injury can alter the GI environment (increase intestinal MC frequency) and food allergic outcome. Consistent with this, corroborative studies in animal model systems have revealed that food exposure via the skin can promote CD4 ^+^ Th2 responses and food allergic reactions ^81– 84^.

Experimental analyses also suggest that inhalation of food allergens can promote food sensitization and reactions ^85, 86^. Dolence *et al*. demonstrated that inhalation of peanut flour allergen in mice leads to the development of food-specific IgE levels and predisposed to food-induced anaphylaxis. Interestingly, draining lymph node-resident T follicular helper (Tfh) cells that produced type-2 cytokines (IL-4 and IL-21) were shown to be the primary drivers of food-specific IgE and food-induced anaphylaxis ^86^. Interestingly, a recent study revealed that indoor dust may act as an adjuvant and play a role in the exacerbation of inhaled food allergen sensitization. Inhaled indoor dust was shown to stimulate airway epithelial innate cytokine production and maturation, and lung type-1 cDCs and co-exposure of indoor dust and food allergens such as peanut led to the development of peanut‐specific Th2 cell differentiation and the accumulation of Tfh cell peanut‐specific IgE production ^85^.

## Intestinal epithelial antigen passages

While food exposure to the skin, particularly in infants, is common, dietary food components (carbohydrates, proteins, lipids, solutes, water) and the resulting digested soluble protein allergens are predominantly absorbed in the SI (duodenum, jejunum, and ileum) and large intestine (colon and rectum) ^87^. The tolerogenic inductive sites including organized lymphoid tissues such as the SI draining mesenteric lymph nodes (MLNs) and the specialized immune cell populations including mucosal DCs and Tregs are dominant within this GI compartment, supporting the concept that the SI is key in the establishment of the tolerogenic response and immune homeostasis ^88, 89^. The mechanism by which the specialized immune cells of the GI tract acquire dietary food antigens is not as well understood. Experimental evidence supports the involvement of several mechanisms in the translocation of food antigens across the SI lumen, including microfold cell-mediated transcytosis, sampling by transepithelial dendrites of mucosal DCs, and paracellular leak ^90, 91^.

Recently, a role for goblet cells (GCs) was identified in the uptake and translocation of GI luminal antigens across the epithelium and presentation to the immune compartment by a process known as GC antigen passages (GAPs) ^92^. GAPs are spatially and temporally regulated and are present in both the SI and the distal colon at steady state ^92, 93^. In the SI, GAP formation and antigen uptake is initiated around day 18 of life, is activated by acetylcholine (ACh) through a muscarinic type 4 receptor (M4AchR)-dependent process, and is maintained throughout adulthood ^94^. SI GAPs are not sensitive to commensal microbiota ^93, 95^; however, they are sensitive to pathogenic infections such as Salmonella ^90^. It is currently postulated that the inhibition of SI GAPs by pathogenic infections is likely a host-dependent response to limit systemic dissemination of pathogenic organisms. Similar to SI GAPs, colonic GAPs are activated by M4AchR-dependent processes; however, colonic GAPs are sensitive to the microbial environment ^94^. The tightly regulated anatomical compartmentalization of GAPs in the SI and colon is likely a mechanism whereby the GI epithelium directs luminal antigen sampling to tune immune development and limit pathogenic antigen exposure and development of inappropriate inflammatory responses ^94, 96^. GAPs appear to deliver GI luminal antigens to LP-antigen-presenting cells such as CD103 ^+^ DCs ^92^ and CX _3_CR1 ^+^ DCs ^92^ and induce T-cell responses ^95^. In the SI, both CD103 ^+^ DCs and CX _3_CR1 ^+^ DCs are capable of acquiring luminal antigen and inducing T-cell responses; however, the frequency of SI GAPs and CD103 ^+^ DC interactions is more dominant. In contrast, colonic CX _3_CR1 ^+^ DCs appear to be the dominant antigen-presenting cell that interact with colonic GAPs and acquire luminal antigens, including commensal bacteria ^95^. These studies lend nicely to the concept that SI GAPs transfer SI soluble protein antigens predominantly to CD103 ^+^ DCs, which contribute to the development of oral tolerance, whereas LI colonic GAPs predominantly interact with CX _3_CR1 ^+^ DCs and transfer microbial antigens and macromolecules for the induction of antigen-specific tolerance to gut bacteria ^94^.

We recently showed that SI GAPs participate in dietary protein antigen uptake ^97^. We showed that in naïve mice, clinically relevant food allergens are acquired by SI GC (MUC2 ^+^) cells. Surprisingly, under food allergic conditions, the SI antigen passage repertoire and frequency were dysregulated. Moreover, the frequency of SI GAPs was significantly greater in the food-allergic mice than in the naïve WT BALB/c mice at steady state. Furthermore, we identified MUC2 ^- ^Rh-Dex ^+^ cells in the SI villus, which were identified as antigen passaging enteroendocrine and Paneth cells, in the SI of food-allergic mice ^91^. These data suggest that under food-allergic conditions, multiple intestinal secretory cell lineages within the SI can acquire and channel food antigens from the apical to basolateral side, which we defined as SI secretory epithelial cell antigen passages (SAPs) ^91^. Mechanistic analyses revealed that SI SAPs were predominantly regulated by the Th2 cytokine IL-13 and not IL-4, via a direct IL-4Rα-STAT6-independent PI3K-CD38-cADPR-dependent process and rapidly channel food antigens directly to FcεR1 ^+^ c-Kit ^+^ ST2 ^high^ mucosal MCs in food-allergic mice. Notably, pharmacological or genetic blockade of IL-13-driven SAP formation protected mice against a food-induced anaphylactic reaction ^91^. Importantly, using a human intestinal organoids (HIOs) model system, we were able to show that both GAPs and SAPs were conserved in human tissue. Collectively, these studies reveal that the food-sensitized state and heightened levels of type-2 cytokines can lead to the reprogramming of the cellular patterning of intestinal epithelial antigen passages and presentation of dietary antigens to different immune compartments. The precise mechanism by which food allergens stimulate SAP formation and passage of antigens to mucosal MCs is currently unknown and under further investigation.

## Molecular mechanisms of food-induced anaphylaxis severity

Food‐induced anaphylaxis leads to a number of symptoms that can affect one or more target organs ^5, 98, 99^. Involvement of either the cardiovascular or the respiratory system constitutes a severe food allergic reaction ^5, 98^, and this may be a result of basophil- and MC-derived mediators inducing pulmonary venous vasodilatation and fluid extravasation, leading to the respiratory and cardiovascular collapse associated with the severe, life-threatening anaphylactic phenotype ^100^. It is uncertain which cellular and molecular pathways directly contribute to this anaphylaxis phenotype. Higher levels of IL-4 and histamine have been reported in the serum of human patients with severe anaphylaxis ^101^, indicating that these molecules may be involved in the expression of the severe disease phenotype. Recently, our group identified an important role for IL-4 in amplifying histamine-induced anaphylaxis responses ^102^. Employing both active and passive models of IgE-mediated anaphylaxis, we showed that IL-4 exacerbated histamine-induced hypovolemic shock in mice and that this was dependent on vascular endothelial (VE) expression of IL-4Rα. Mechanistic analyses revealed that IL-4 and histamine induced ABL1 activation in human VE cells and that VE barrier dysfunction was ABL1 dependent. The development of severe IgE-mediated hypovolemia and shock required VE-restricted ABL1 expression. Treatment of mice with a history of food-induced anaphylaxis with the ABL kinase inhibitor imatinib protected the mice from severe IgE-mediated anaphylaxis. Collectively, IL-4 amplifies IgE- and histamine-induced VE dysfunction, fluid extravasation, and the severity of anaphylaxis through a VE-IL-4Rα/ABL1-dependent mechanism. These findings suggest that ABL1 kinase could be a potential therapeutic target for preventing IgE-mediated anaphylaxis. Considering that tyrosine kinase inhibitors (TKIs), such as imatinib, can target both endothelial cells and c-Kit-mediated MC development and survival, targeting ABL1 could provide a “double hit” to potently attenuate IgE-mediated responses. Interestingly, a recent study showed imatinib to be efficacious in severe refractory asthma patients, with decreased MC numbers and asthma symptoms ^103^.

## Advances in food allergy treatment

Epinephrine remains the first line of acute treatment for food-induced anaphylactic reactions, acting through the α1/β1/β2-adrenergic receptors to temper the pathophysiologic response ^2– 4, 7^. Along with epinephrine, H1- and H2-antihistamines are also used to treat anaphylactic reactions ^104^, and omalizumab, an anti-IgE monoclonal antibody, has been shown to be effective in patients with idiopathic anaphylaxis with IgE involvement ^2, 105^. Recently, there has been increasing attention in the usage of oral immunotherapy (OIT) as a new approach to treating IgE-mediated food allergies. OIT involves oral exposure of gradual increasing doses of the eliciting allergen under close medical supervision, with the starting dose lower than what typically may trigger an allergic reaction and performing dose escalations (approximately every 2 weeks) until one achieves a maintenance dose (typically about 4–6 months), and this maintenance dose is continued indefinitely. Several clinical trials have demonstrated good safety profiles, decreased allergen-specific IgE, and effectiveness in raising the threshold of allergen needed to trigger an allergic reaction ^106– 110^.

In an exciting development for peanut-allergic individuals, AR101 (Palforzia), an investigational peanut protein biologic, has recently been approved for peanut OIT treatment to reduce allergic reaction incidence and severity in peanut-allergic patients aged 4–17 years old. In the Peanut Allergy Oral Immunotherapy Study of AR101 for Desensitization (PALISADE NCT02635776), a randomized, double-blind, placebo-controlled, phase III trial, OIT using AR101 significantly lowered reactivity and decreased severity of symptoms in patients 4–17 years of age ^107^. A total of 67% of patients who received AR101 were able to tolerate the 600 mg exit challenge dose without any dose-limiting symptoms, as compared to 4% of the placebo group. At the exit food challenge, 25% and 5% of patients treated with AR101 showed moderate and severe symptoms, respectively, while 59% and 11% of those receiving placebo showed moderate and severe symptoms, respectively ^107^. While these findings are very encouraging, there are several limitations in this study, preventing broader applicability. Firstly, patients 18–55 years of age upon treatment with AR101 did not show significant protection at exit food challenge. Furthermore, the study selected only patients who showed dose-limiting symptoms to up to 100 mg of peanut, thus excluding approximately 50% of peanut-allergic patients ^107, 111^. Finally, AR101 efficacy or safety could not be established in patients with severe co-morbidities, since patients with severe or poorly managed asthma were excluded because of safety considerations. Some of these limitations are being addressed in ongoing peanut OIT trials: NCT03201003, NCT02993107, and NCT03292484. Other questions concerning optimization of OIT, such as optimal dose and duration of maintenance and the sustainability of this state of desensitization or unresponsiveness, are also currently under investigation.

Supplementation with probiotics for the prevention or treatment of food allergy has also been an area of active research. Previous studies have reported that supplementation of extensively hydrolyzed casein with *Lactobacillus rhamnosus* ^112, 113^, but not *Lactobacillus casei* and *Bifidobacterium lactis* ^114^, has been shown to accelerate tolerance induction in cow’s milk-allergic individuals. *Lactobacillus rhamnosus* supplementation is known to promote the expansion of tolerogenic butyrate-producing bacterial strains (e.g. *Lachnospiraceae* and *Ruminococcaceae*) ^115^, leading to speculation that probiotics can expand tolerance-promoting microbes and drive immunological tolerance to foods. A randomized placebo-controlled trial designed to evaluate the effect of coadministration of a probiotic and peanut OIT (PPOIT) demonstrated that PPOIT was effective in inducing possible sustained unresponsiveness and immune changes in peanut-allergic children ^116^. A 4-year follow up study to assess long-term outcomes revealed that PPOIT provided long-lasting clinical benefit and persistent suppression of the allergic immune response to peanut ^117^.

Despite the clinical success of OIT achieving average desensitization rates of 80–85%, the underlying cellular and molecular processes that mediate OIT desensitization are not yet fully elucidated ^14, 118^. Rush desensitization (DS), a clinical protocol often used to manage drug allergies, has been shown to rapidly render allergic individuals temporarily hyporesponsive to the antigen and permits the individuals to tolerate eliciting drug exposure. Similar to OIT, DS involves exposure of an individual to increasing doses of the eliciting antigen; however, DS is over a short interval of time, typically with minutes or hours between doses. Oral DS has been successfully used to allow patients to tolerate the first dose of immunotherapy regimens for certain food allergies ^119, 120^. The temporary hyporesponsiveness to the eliciting antigen is thought to be mediated by MC desensitization, where repeated low-dose antigen exposure causes gradual and limiting release of MC-derived mediators until achieving cell exhaustion and mediator depletion, leading to a diminished IgE-FcεRI-dependent MC response. Anecdotal clinical evidence demonstrating that short-term discontinuation of OIT treatment can lead to responsiveness in some tolerant OIT individuals and the observed reduced allergen-specific MC and basophil degranulation in individuals during OIT in principal support the concept of MC sensitization involvement ^121, 122^.

An alternative hypothesis is that OIT desensitization is mediated by the development of allergen-specific IL-10 ^+^ and TGF-β ^+^ Tregs or exhaustion/deletion of memory allergen-specific CD4 ^+^ Th2 cells, leading to immunological and clinical tolerance ^123– 125^. A recent study revealed that patients who underwent OIT had increased frequency of peanut-specific Tregs. Interestingly, the patients with sustained unresponsiveness had significant hypomethylation at *FoxP3* CpG sites in antigen-induced Foxp3 ^+^ Tregs, while patients unable to maintain sustained unresponsiveness had hypermethylation, suggesting that epigenetic changes made during or after OIT could contribute to OIT-induced sustained unresponsiveness ^126^. In mice, milk OIT increased SI levels of tolerogenic cytokines IL-10 and TGF-β ^125^. Furthermore, GATA3 hypermethylation in CD4 ^+^ Th2 cells and FOXP3 hypomethylation in Tregs have been observed in epicutaneous immunotherapy-treated mice ^127^. Recently, Wambre *et al*. identified a terminally differentiated allergen-specific memory Th2 cell population, termed TH2A cells ^128^. TH2A cells co-express CRTH2, CD49d, and CD161 and are potent producers of the cytokines IL-5 and IL-9 ^128^. Interestingly, OIT in peanut-allergic individuals significantly decreased the frequency of TH2A cells, suggesting that OIT may induce exhaustion/deletion of memory allergen-specific Th2 cells ^128^. Understanding the immunological processes that drive OIT desensitization and clinical tolerance will be critical for the development and optimization of OIT biologics and protocols.

## Summary

It is becoming increasingly clear that a dynamic and complex interplay among dietary components, environmental stimuli, microbiota, and host immunity is required to develop and sustain the unresponsiveness to dietary food antigens. Dysregulation of these key processes leads to a breakdown of oral tolerance and allergic sensitization. A deeper understanding of the molecular and cellular mechanisms that underlie the breakdown of tolerance will be critical in guiding precision medicine approaches and directing better diagnosis, management, and safer and more efficacious treatment options for patients of food allergy and anaphylaxis.
